# Repeated Pelvic Radiographs in Infants, After Harness Treatment for Developmental Dysplasia of the Hip, Carry Very Low Radiation Risk

**DOI:** 10.1007/s43465-021-00438-x

**Published:** 2021-06-30

**Authors:** Elizabeth Vogel, Thomas Leaver, Fiona Wall, Ben Johnson, Michael Uglow, Alexander Aarvold

**Affiliations:** 1 Southampton Medical School, Southampton University, Southampton, UK; 2 Medical Physics Department, University Hospital Southampton, Southampton, UK; 3 Paediatric Orthopaedics, Southampton Children’s Hospital, Southampton, UK

**Keywords:** DDH, X-rays, Radiation, Malignancy, Risk

## Abstract

**Objective:**

There are no data on the effect of X-Ray irradiation to the vulnerable pelvic organs of babies during DDH follow-up. This study aims to calculate, for the first time, the radiation exposure to infants during follow-up for DDH harness treatment, and thus quantify the lifetime risk of malignancy.

**Methods:**

Patients who had completed 5 years’ follow-up following successful Pavlik harness treatment were identified from the hospital DDH database. The radiation dose was extracted from the Computerised Radiology Information System database for every radiograph of every patient. The effective dose (ED) was calculated using conversion coefficients for age, sex and body region irradiated. Cumulative ED was compared to Health Protection Agency standards to calculate lifetime risk of malignancy from the radiographs.

**Results:**

All radiographs of 40 infants, successfully treated in Pavlik harness for DDH, were assessed. The mean number of AP pelvis radiographs was 7.00 (range: 6–9, mode: 7). The mean cumulative ED was 0.25 mSv (Range: 0.11–0.46, SD: 0.07). This is far lower than the *annual* ‘safe’ limit for healthcare workers of 20 mSv and is categorised as “Very Low Risk”.

**Conclusion:**

Clinicians involved in the treatment DDH can be re-assured that the cumulative radiation exposure from pelvic radiographs following Pavlik harness treatment is “Very Low Risk”. Whilst being mindful of any radiation exposure in children, this study provides a scientific answer that help addresses parental concerns.

## Introduction

Developmental dysplasia of the hip (DDH) is common, with up to 1% of newborns treated in most countries [1–3]. In order to make a diagnosis, ultrasound scans (USS) are used to confirm clinical suspicion, after which abduction splinting is used in the first instance for management [4, 5]. The Pavlik harness is the most widely used, with high success rates [4], though other fixed and dynamic splints are utilised [6–8].

All medical and surgical interventions carry a degree of risk. With harness use, specific risks include avascular necrosis of the femoral head, femoral nerve palsy and failure to achieve reduction [9]. As such, both clinical and radiological monitoring of progress is essential [10]. Whilst initial diagnosis and monitoring are via USS, as the infant grows and the ossific nucleus develops, the clarity of hip morphology seen on USS decreases [3]. Radiographs become essential to monitor further development of the infant hips, usually beyond the age of 6–12 months. The duration of follow-up varies across hospitals and countries, ranging from discharging in infancy to ongoing surveillance to skeletal maturity [11].

Whilst radiographs of the hips are integral to follow-up, the X-ray radiation exposure carries a risk, particularly in the paediatric population [12]. The radio-sensitive organs located within and around the pelvis, such as the testes or ovaries, are particularly vulnerable. Thus, age and body region imaged make DDH patients a particularly susceptible group to radiation exposure. The ionising, and thus potentially dangerous, effect X-rays can have on tissues is well documented, for example, in the rate of malignancies in radiation workers in the past [13]. Whilst the true implications of imaging-related malignancies are not completely understood, clinical practice is constantly evolving to minimise risk [12, 14, 15]. There are believed to be 700 new cases of cancer each year in the UK that can be directly linked to diagnostic radiographs [16]. This is particularly concerning in a paediatric setting for two major reasons. First, the younger growing tissues are more vulnerable to the harmful radiation. Second, children have a longer lifetime ahead of them during which, following any radiation damage to the genetic material, there is more time to manifest as malignancies [13, 16–20].

Concern over the risk associated with irradiating their baby’s pelvic region means parents and guardians will commonly question the necessity of the radiographs. This is a valid concern of parents who, by agreeing to the radiographs, are consenting to radiation exposure to their child’s vulnerable developing pelvic organs. The senior author is asked this question by parents in his practice at least every fortnight. Yet, to date, the risk of such radiation exposure has never been explored.

Relative risk of medical radiation exposure can be quantified by calculating the effective dose (ED) of radiation [21]. The ED takes into consideration the biological effect on the area undergoing imaging, so this is highly relevant in this patient population. As an example, the specific conversion co-efficient for ED from imaging a pelvis is approximately 17 times higher than imaging a lower limb [17]. So, despite a low radiation dose being used, the biological effect can be great. The relative risk per unit dose over a lifetime is also age-dependent, which is also a highly relevant factor in this patient population. This study aims to calculate the lifetime risk of malignancy from radiation exposure to the pelvis in this young patient population, in relation to pre-existing Public Health England reference values. Having a quantified value for radiation risk will allow a more definitive answer to be provided to parents and guide optimal follow-up strategies.

## Materials and Methods

This work was carried out in a tertiary referral unit for DDH. Approximately 50–100 infants per year in our institution are diagnosed with DDH through the Newborn and Infant Physical Examination (NIPE) national selective USS screening programme [22], and treated in Pavlik harness. This equates to 7 in 1000 live births, with a Pavlik harness success rate of 95% [4]. These infants are routinely followed up to 5 years of age, with the first radiograph occurring around 12 months of age, then 4–6 monthly until 24 months old and annually to 5 years of age.

The last forty sequential patients to complete 5 years of follow-up were selected for inclusion. Patients for whom Pavlik harness treatment failed (and thus progressed to surgical intervention) were excluded, as were any who had not completed the full 5 years of follow-up. Each patient was allocated a unique identification number, selected by a random number generator (Excel 2007), against which all demographic and radiation data were recorded. This was done to ensure no patient identifiable information was included within the dataset. Ethical approval was gained using the Ethics and Research Governance Online system (Ref. 42995).

The dates, type and number of radiographs for each patient were collected. Any radiological imaging that was not related to DDH was not included as, despite having an impact on their cumulative radiation exposure, the aim of this study was to quantify the DDH related radiation risk. The numerous USS that, the patients underwent were not included, as USS involves non-ionising waves and does not add to any radiation exposure. At this institution, genital shields are not used for the first radiograph but are used for all subsequent images. Practice variation exists across hospitals regarding use of gonadal shields, with scatter and reflection from the shields potentially even causing an *increase* in radiation dose onto the genitals [23, 24].

**Fig. 1:**
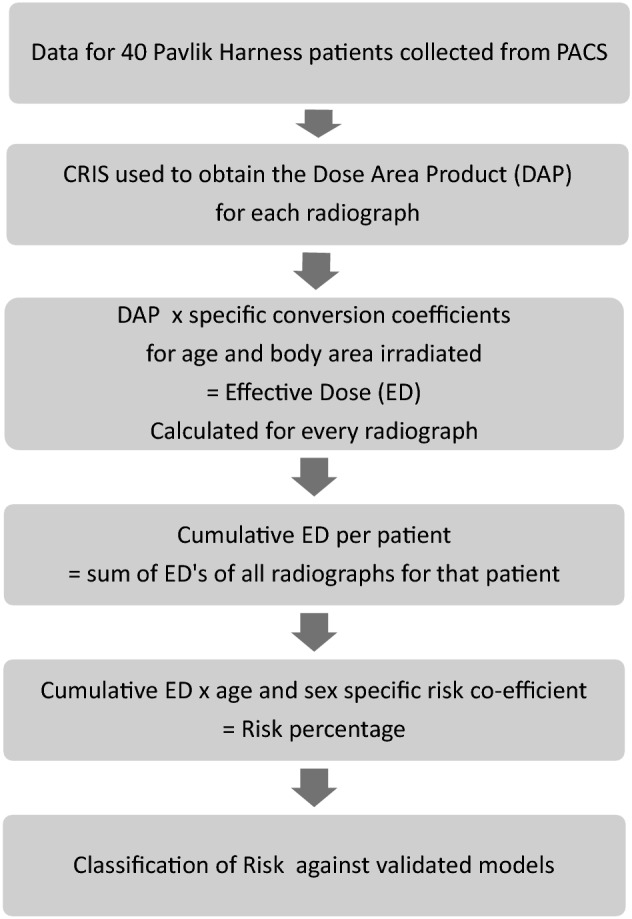
Flow chart to summarise the calculations used to quantify radiation exposure, from first radiograph to lifetime risk. *PACS* Picture Archiving and Communication System, *CRIS* Computerised Radiology Information System, *DAP* Dose Area Product, *ED* Effective Dose

The dose area product (DAP), measured in Gy/cm^2^, for every radiograph was extracted from the hospital Computerised Radiology Information System (CRIS). The Effective Dose (ED), measured in millisieverts (mSv), is the unit by which radiation risk is quantified [21]. The ED for every radiograph was individually calculated from the DAP using specific conversion coefficients for the area of the body irradiated [18]. The specific cumulative ED for each child was compiled. This process is outlined in Fig. 1.

Cumulative ED was compared to established risk models for medical imaging, taking into account the child’s age and sex at the time of the radiograph [19]. This enabled the lifetime risk of malignancy to be quantified, placing each child into a Public Health England (PHE)-validated risk category. The cumulative ED was also compared to the annual UK background radiation exposure and the ‘safe’ limits for workers exposed to radiation [20]. Statistics were performed on SPSS (IBM, 2020).

## Results

Of the 40 sequential patients included, 32 were female and 8 male, reflecting the standard gender discrepancy of DDH [25]. Harness treatment for all forty patients was commenced during the 2014 calendar year, all aged less than 12 weeks at harness application, with 5 years’ follow-up completed during the 2019 calendar year. There was a prescriptive follow-up regime, with the first radiograph occurring at 12 months of age and each patient with a normal clinical and radiographic examination of their hips being discharged at 5 years of age. Most children received seven pelvic radiographs during this time, with the range being six to nine. Increased frequency of radiographs occurred if there was any doubt as to the presence of residual dysplasia.

The DAP of the radiographs ranged from 0.003 to 0.081 Gy·cm^2^. The range is related to variation between individual patients, in terms of body habitus and size. There was a slight positive skew, with a median DAP of 0.036 Gy·cm^2^. The ED for each radiograph, which accounted for patient age and organ sensitivity, ranged from 0.011 to 0.151 mSv. The maximum cumulative ED that a child was exposed to was 0.463 mSv, with the minimum being 0.107 and a mean of 0.255 mSv (SD 0.07). This level of radiation exposure, when categorised according to validated PHE reference tables [26], carries a lifetime risk of malignancy of 0.002% from this medical imaging. This equates to 1 in 50,000 and is classified as “Very Low Risk” (Table 1).

**Table 1:** Summary of lifetime risk from radiation exposure

Mean cumulative effective dose (mSv)	Mean percentage risk (%)	Risk per thousands
0.255	0.002	1 in 50 000

**Fig. 2:**
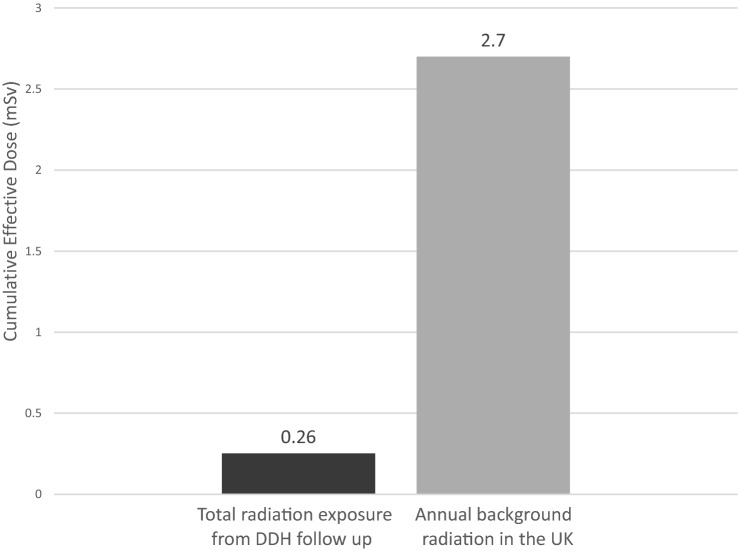
Comparison of X-ray exposure from DDH follow-up radiographs over 5 years to the annual background radiation in the UK [20]

To provide further context to these figures, the cumulative ED was compared to relevant well-understood radiation exposure levels, specifically annual background radiation exposure in the UK and the annual ‘safe’ upper limit of radiation exposure for an employee in healthcare. The *annual* background radiation from living in the UK is 2.7 mSv, which is 11 times greater than the mean *cumulative* ED from the DDH follow-up radiographs up to 5 years of age (Fig. 2). Thus, the additional risk associated is far smaller than the risk from annual background radiation exposure. The mean *cumulative* ED from the DDH radiographs (0.255 mSv) is 79 times lower than the maximum ‘safe’ *annual* exposure for healthcare workers (20 mSv) and is 317 times lower than the permitted ‘safe’ cumulative exposure over the same timeframe (80 mSv).

## Discussion

This study has found that the radiation exposure to infants during routine follow-up of DDH after Pavlik harness treatment carries ‘Very Low Risk’ of lifetime malignancy [17, 26]. Whilst the risk can never be zero, the figures calculated in this study can be used to reassure worried parents who question the safety and necessity of these follow-up radiographs. Furthermore, it should reassure those in community medicine that, if a single diagnostic pelvic radiograph is indicated, it carries very low risk even in a young child.

The comparison of cumulative ED from all DDH radiographs over full follow-up, to that of *annual* background radiation is stark. The DDH follow-up radiographs had a *cumulative* ED that was 11 times smaller than *annual* background radiation in the UK. To add further context, the baseline cancer risk for a childhood malignancy in the UK is 1 in 500, or 0.2% [27]. The 0.002% *lifetime* risk of malignancy related to these DDH follow-up radiographs therefore corresponds to an additional 1/100th of this. Furthermore, the reader will have annual ‘safe’ limits of radiation exposure in their capacity as a healthcare worker, which are almost 80 times higher than the cumulative radiation exposure to the children that has been quantified in this study.

There are always improvements to be made in the safety of X-ray technology, as shown by studies looking at the impact of older forms of X-rays. In particular, the incidence of leukaemia in radiologists before the 1950s was significant [13]. This has been dramatically improved, to zero risk of malignancy, from medical imaging in radiologists who graduated after the 1940s [28]. This difference in malignancy in radiologists demonstrates how improvement in radiation safety can make differences in health quality in the future. Thus, it is crucial to always strive for safer procedures, especially for the particularly vulnerable paediatric population.

The importance of radiographs within orthopaedics is ever increasing. They are used both diagnostically, including computed tomography (CT), and as part of treatment in the context of intra-operative fluoroscopy [29]. Within paediatric orthopaedics, numerous conditions encompass follow-up which involves radiographic imaging. The risk associated with routine follow-up of patients with Perthes Disease or hip surveillance in Cerebral Palsy is ground for future research [30, 31]^.^ Whilst radiographs are so important for complete assessment, this study supports that the radiation principles ‘As Low As Reasonable Achievable (ALARA)’ are being adhered to for DDH follow-up [32].

There are limitations within the calculations used in this study. The co-efficient conversion table used to calculate risk has the age of patients split into categories rather than discrete ages. The categories are ‘below 1 year old’, ‘1–5 years old’ and ‘five and above’ [18]. Thus, all patients in this study after their first radiograph fell into the 1- to 5-year-old category. It is likely that a 2-year-old carries a higher risk than a 5-year-old, but the specific conversion coefficients for each year of age do not exist. A further limitation was that the dose area product (DAP) for some radiographs on the CRIS database was recorded as 0.00 Gy·cm^2^. This is a recording error rather than a rounding error. Using the recorded value of zero would have negatively skewed the cumulative ED, therefore, to provide the most accurate value, the mean value of the other radiographs for that specific patient was used for any missing DAP result. Using an individualised average, it minimised the deviation from the true value, but may have reduced the accuracy.

This study was limited to forty patients, in keeping with a similar study on radiation exposure in paediatric limb deformity [33]. Whilst there were some variations in the ED between the patients in this study, this patient group was more homogenous than the limb deformity group, and the range of cumulative ED was minimal. The cumulative ED of all patients fell comfortably within the same risk category, thus including more patients is highly unlikely to alter the conclusions. This study has not however included infants who had surgery for DDH, for whom the radiation exposure is likely to be higher. This is the subject of ongoing analysis and will undoubtedly involve higher radiation exposure.

This study has quantified, for the first time in infants treated for DDH, the radiation risk from X-ray exposure of pelvic radiographs. The lifetime risk of malignancy is ‘Very Low Risk’ from routine follow-up for Pavlik harness-treated infants. This information can be used to reassure parents and guardians who, very reasonably, may question the necessity of the radiographs for their infants.
